# Effects of Acute Iso- and Hypocaloric Carbohydrate Restriction on Liver Fat and Glucose and Lipid Metabolism

**DOI:** 10.1210/clinem/dgaf382

**Published:** 2025-07-02

**Authors:** Amalie London, Amanda Schaufuss, Michal Považan, Marie-Louise Dichman, Jasmin Merhout, Carsten Dirksen, Sten Madsbad, Hartwig Roman Siebner, Annemarie Lundsgaard, Andreas Mæchel Fritzen, Bente Kiens, Kirstine Nyvold Bojsen-Møller

**Affiliations:** Department of Endocrinology, Copenhagen University Hospital Hvidovre, Hvidovre 2650, Denmark; Department of Nutrition, Exercise and Sports, The August Krogh Section for Human and Molecular Physiology, Faculty of Science, University of Copenhagen, Copenhagen 2100, Denmark; Department of Endocrinology, Copenhagen University Hospital Hvidovre, Hvidovre 2650, Denmark; Department of Biomedical Sciences, Faculty of Health and Medical Sciences, University of Copenhagen, Copenhagen 2200, Denmark; Danish Research Centre for Magnetic Resonance (DRCMR), Centre for Functional and Diagnostic Imaging and Research, Copenhagen University Hospital Hvidovre, Hvidovre 2650, Denmark; Department of Endocrinology, Copenhagen University Hospital Hvidovre, Hvidovre 2650, Denmark; Danish Research Centre for Magnetic Resonance (DRCMR), Centre for Functional and Diagnostic Imaging and Research, Copenhagen University Hospital Hvidovre, Hvidovre 2650, Denmark; Department of Endocrinology, Copenhagen University Hospital Hvidovre, Hvidovre 2650, Denmark; Department of Clinical Medicine, Faculty of Health and Medical Sciences, University of Copenhagen, Copenhagen 2200, Denmark; Department of Endocrinology, Copenhagen University Hospital Hvidovre, Hvidovre 2650, Denmark; Department of Clinical Medicine, Faculty of Health and Medical Sciences, University of Copenhagen, Copenhagen 2200, Denmark; Danish Research Centre for Magnetic Resonance (DRCMR), Centre for Functional and Diagnostic Imaging and Research, Copenhagen University Hospital Hvidovre, Hvidovre 2650, Denmark; Department of Clinical Medicine, Faculty of Health and Medical Sciences, University of Copenhagen, Copenhagen 2200, Denmark; Department of Neurology, Copenhagen University Hospital Bispebjerg and Frederiksberg, Copenhagen 2200, Denmark; Department of Nutrition, Exercise and Sports, The August Krogh Section for Human and Molecular Physiology, Faculty of Science, University of Copenhagen, Copenhagen 2100, Denmark; Department of Biomedical Sciences, Faculty of Health and Medical Sciences, University of Copenhagen, Copenhagen 2200, Denmark; Department of Nutrition, Exercise and Sports, The August Krogh Section for Human and Molecular Physiology, Faculty of Science, University of Copenhagen, Copenhagen 2100, Denmark; Department of Endocrinology, Copenhagen University Hospital Hvidovre, Hvidovre 2650, Denmark; Department of Clinical Medicine, Faculty of Health and Medical Sciences, University of Copenhagen, Copenhagen 2200, Denmark

**Keywords:** lipid metabolism, glucose metabolism, low-carbohydrate, mixed meal test, MASLD (metabolic dysfunction–associated steatotic liver disease)

## Abstract

**Context:**

Liver fat is reduced within days after a low-carbohydrate diet substituted with fat to maintain isocaloric conditions.

**Objective:**

Investigate the effect of matched carbohydrate restriction during isocaloric and hypocaloric conditions on liver fat and postprandial glucose and lipid metabolism.

**Methods:**

Crossover randomized clinical trial at a research unit. Participants included 15 people with overweight/obesity (body mass index [BMI] 32.5 [31-34] kg/m^2^, median [IQR]). There were 3 dietary interventions: (1) 2 days of isocaloric control diet (CON), (2) 2 days of CON followed by 2 days of carbohydrate restriction (∼60 g/day) during very-low–calorie conditions (VLCDs), and (3) 2 days of CON followed by 2 days of isocaloric conditions with low carbohydrate (∼60 g/day), high-fat (LCHF) diet. Liver fat was measured using ^1^H-magnetic resonance spectroscopy.

**Results:**

Liver fat was −16% (−34; 4) (median [IQR]) after LCHF relative to after CON (*P* = .020), but did not differ between VLCD and CON. Fasting plasma concentrations of triacylglycerol, glucose, and insulin were lower after both LCHF and VLCD than after CON. However, postprandial plasma glucose concentrations were higher and insulinogenic index lower after both LCHF and VLCD.

**Conclusion:**

Two days of LCHF led to lower liver fat, which was not observed after VLCD. This demonstrates the dynamic regulation of liver fat and the beneficial role of substituting carbohydrates with fat to maintain energy provision. Both LCHF and VLCD had positive effects on fasting parameters for glucose metabolism; however, both diets impaired early β-cell response resulting in deterioration in glucose handling during the meal test.

Intrahepatic triacylglycerol content (liver TG) is associated with several metabolic disturbances, including insulin resistance, impaired glucose tolerance, and dyslipidemia (1, 2). Simultaneously, excess liver TG is associated with increased risk of progression to irreversible liver disease and cardiovascular disease (3, 4). However, liver TG content has also been demonstrated to change markedly in response to acute dietary changes (5-8), suggesting that a given level of liver TG besides affecting metabolism by itself is also a reflection of the current metabolic state and the recent diet of the individual. Hence, the lowering of liver TG content is likely to be an important contributor to the improvements in glucose metabolism observed within days of calorie restriction (eg, after gastric bypass surgery) (9, 10). Furthermore, liver TG content is reduced by 30% within 2 to 7 days after initiation of a hypocaloric diet in individuals with obesity and/or type 2 diabetes (5, 6). During a low-calorie diet, the reduced energy intake is achieved by restricting the absolute intake of all macronutrients; carbohydrates, protein, and fat. However, emerging evidence suggests that manipulating dietary macronutrient composition without restricting energy may also significantly impact liver TG content. Accordingly, low-carbohydrate, high-fat diets are efficient in lowering liver TG content and improve glycemic control in individuals with obesity and/or type 2 diabetes (11-18). The precise role of carbohydrate restriction for the beneficial effects of low-calorie diets and low-carbohydrate, high-fat diets is challenging to unravel, and it is still uncertain if liver TG is lowered by carbohydrate restriction per se, or whether there is a beneficial effect of substituting carbohydrates with fat.

We therefore conducted a crossover randomized clinical trial including males and postmenopausal females with overweight and obesity. The trial aimed to assess liver TG content and glucose and lipid metabolism after 2 days of matched absolute carbohydrate restriction (approximately 60 g/day) either under isocaloric (substituting dietary carbohydrates with fat), or hypocaloric conditions compared with 2 days of control diet resembling habitual food intake. We chose an acute diet intervention to minimize confounding influences from weight changes. We hypothesized that liver TG content would be lower after both iso- and hypocaloric carbohydrate–restricted diets than after the control diet (primary outcome). Additionally, we investigated the distinct whole-body metabolic responses to the diets in the overnight fasted state as well as after reintroducing dietary carbohydrates during a mixed meal test.

## Materials and Methods

Inclusion criteria were males and females aged 35-75 years with a body mass index (BMI) of 25 to 40 kg/m^2^ and HbA_1c_ (<48 mmol/mol). Premenopausal females were excluded to avoid the metabolic impact of differences in the phases of the menstrual cycle. Further exclusion criteria were smoking, anemia, thyrotropin outside reference range, medication known to influence glucose metabolism or appetite regulation, contraindications for ^1^H-magnetic resonance spectroscopy [^1^H-MRS] scanning, food allergies (including lactose and gluten intolerance), vegetarian/vegan diet or following specific dietary plans, alcohol consumption >84/168 g/week (for females/males, respectively), daily use of nicotine products, >5 kg weight change within the last 3 months, previous bariatric surgery, or high risk of fibrosis in the liver (estimated by FIB-4 score > 3.25) (19). The study was approved by the scientific-ethical committee of the Capital Region of Denmark (H-22035189) and carried out in accordance with the Declaration of Helsinki. The study was preregistered at clinicaltrials.gov (NCT05643521).

Participants were recruited between January 2023 and September 2023, with experiments conducted from February 2023 to October 2023.

### Study Design

The study was designed as a 3-arm crossover randomized clinical trial; Participants completed 3 dietary interventions, each separated by a minimum of 1 week: (1) 2 days of a conventional diet (CON) resembling habitual food intake, (2) 2 days of CON followed by 2 days of a carbohydrate-restricted very-low calorie diet (VLCD), and (3) 2 days of CON followed by 2 days of an isocaloric carbohydrate-restricted, high-fat diet (LCHF). Participants were randomly assigned to the intervention order using block randomization with sealed envelopes.

### Metabolic Test Day

Following each of the 3 dietary interventions, the participants completed the metabolic test day. On the metabolic test day, participants arrived at 7 Am by passive transportation after an overnight fast (approximately 12-hours) and after having abstained from vigorous physical activity for 48 hours. Body composition was determined using bioelectrical impedance analysis (SECA mBCA 515, MFBIA; SECA, Germany). Afterwards, a single-voxel ^1^H-MRS scan was performed to determine liver TG content. After the ^1^H-MRS scan, participants rested in a supine position in a hospital bed for 30 minutes after which whole-body resting metabolic rate and respiratory exchange ratio (RER) were determined by indirect calorimetry (Vyntus CPX Canopy, Vyaire Medical, USA) with measurements obtained for 10 minutes, and breath by breath data collected for the last 7 minutes. A catheter was then inserted into an antecubital vein for blood sampling. A liquid mixed meal was consumed evenly over 10 minutes (400 mL Nutricia: 2520 kJ containing in total 74 g of carbohydrate [49 energy-%, E%], 24 g of protein [16E%], and 23 g of fat [35E%]). For evaluation of gastric emptying, 1000 mg of crushed paracetamol was added to the first 100 mL of the liquid meal. Serial measurements of metabolic rate, substrate utilization, and blood samples were obtained at regular intervals from initiation of the meal and for 240 minutes. At time 240 minutes, participants were served an ad libitum mixed meal (pasta Bolognese; 533 kJ/100 g; 53E% carbohydrate, 14E% protein, and 33E% fat). The participants were instructed to eat until pleasantly satiated. Drinking 175 mL of water was allowed with the meal. The meal was weighed before and after ingestion to estimate ad libitum food intake. Three participants expressed dissatisfaction with the taste of the ad libitum meal and were not included in this part of the analysis.

### Dietary Interventions

For CON and LCHF diets, the daily energy requirement for each participant was determined by first estimating their basal metabolic rate using a modified Harris–Benedict equation (20). This basic metabolic rate was then adjusted by an estimated physical activity level (PAL), derived from a detailed questionnaire assessing each participant's habitual physical activity pattern.

Diets were fully delivered to the participants, and they were prohibited from consuming any additional food or beverages, or consuming alcohol. Black coffee and tea were restricted to 4 cups per day. VITAKOST software (Denmark) was used to determine the macronutrient composition of the participants habitual diet and to design the intervention diets.

### Plasma and Serum Analysis

Blood samples were drawn twice before meal initiation and at regular intervals postprandially (−10, 0, 15, 30, 45, 60, 90, 120, 180, 240 minutes). For plasma collection, blood was sampled into EDTA-coated tubes (glucose, triacylglycerol (TG), fatty acids (FAs), insulin, C-peptide) or lithium-heparin–coated (fasting cholesterol profile) tubes and immediately centrifuged. Blood samples for serum paracetamol analysis were collected into clot activator tubes and left to coagulate at room temperature for 30 minutes before centrifugation. Plasma glucose concentrations were measured at bedside using the glucose oxidase method (YSI model 2500 STAT Plus; YSI, Yellow Springs, USA). Fasting plasma concentrations of total cholesterol, high-density lipoprotein (HDL) cholesterol, very-low-density lipoprotein (VLDL)-TG, alanine aminotransferase (ALAT), and aspartate aminotransferase (ASAT) were measured on the metabolic test day on a Cobas 6000 (Roche Diagnostics, GmbH, Germany) and for platelets on a Sysmex 1000 XN (Sysmex, Germany). Low-density lipoprotein (LDL) cholesterol concentrations were calculated using the Friedewald equation (total cholesterol-HDL cholesterol – [plasma TG/5]). The rest of the plasma and serum samples were stored at −80 °C or −20 °C until batch analysis. Plasma concentrations of insulin and C-peptide were measured by Immulite 2000 (Siemens Healthcare, Germany). Plasma β-hydroxybutyrate concentration was measured by a colorimetric method (Sigma-Aldrich, USA) using Multiskan FC (Thermo Scientific, USA). Plasma concentrations of FA (NEFA C kit, Wako Chemicals GmbH, Neuss, Germany), glycerol (Randox, Crumlin, UK), and TG (GPO-PAP kit, Roche Diagnostics, Germany) were measured using enzymatic colorimetric methods on an autoanalyzer (Pentra C400 analyzer, Horiba, Japan).

### ^1^H-Magnetic Resonance Spectroscopy of the Liver

Fat content of the liver was measured by single-voxel ^1^H-MRS using a Philips Achieva 3-Tesla scanner (Philips Healthcare, Best, Netherlands). The voxel was placed on a spoiled gradient echo (2D M-FFE) image, carefully avoiding large vessels and bile ducts. 3D reference screenshots ensured consistent voxel placement for each subject. Automatic first- and second-order shimming was performed, using the vendor-supplied shimming routine. Single-voxel spectra were acquired from a 16 mL volume (2.5 × 2.5 × 2.5 cm^3^) using the PRESS sequence with following parameters; TR = 2 seconds, TE = 29 ms, BW = 2 kHz, 1024 samples, 32 water-suppressed spectra, 32 water-unsuppressed spectra. ^1^H-MRS spectra were quantified with LC-Model using automated pipeline in Matlab (MATLAB 2020b, The MathWorks, Inc., Natick, MA, USA). Phase and frequency alignment and eddy current correction were done before signal averaging, and motion-corrupted spectra were removed using the FID-A toolkit (21). Fat fractions were estimated as previously reported (22).

A blinded researcher conducted data analysis, excluding failed scans after quality assurance. Statistical analysis included data from the 10 participants without failed scans.

### Calculations

Fasting concentrations were calculated as the mean of 2 basal blood samples. HOMA calculator (available at https://www.rdm.ox.ac.uk/about/our-facilities-and-units/DTU/software/homa) was used for calculating HOMA2-IR and HOMA-β, using fasting concentrations of plasma glucose and C-peptide. The FIB-4 score was calculated as (age × ASAT)/(platelets × (ALAT^0.5^)). RER was calculated as RER = VCO_2_/VO_2_. Resting energy expenditure (kJ/day) was calculated as resting energy expenditure = (3.941 × VO_2_) + (1.11 × VCO_2_) × 1.44 × 4.1841. Area under the curve (AUC) was calculated using the trapezoid rule, with total AUC (tAUC) representing the total area, incremental AUC (iAUC) as the area above fasting values, and decremental AUC (dAUC) as the area below fasting values. Insulinogenic index (IGI) (C-peptide_30_ − C-peptide_fasting_)/(glucose_30_ − glucose_fasting_) was calculated as an estimate of insulin secretion relative to the increment in glucose concentration. Disposition index, a measure for the β-cells ability to compensate for insulin resistance, was calculated as disposition index = IGI × (1/HOMA2-IR). Insulin clearance was estimated as the fasting plasma C-peptide/fasting plasma insulin. Changes in metabolic flexibility was evaluated as Δwhole-body carbohydrate oxidation rate from basal to 45 minutes after the meal intake (ΔCHO ox).

### Statistical Analyses

Data are presented as means ± SE unless otherwise specified. Data that exhibited a non-normal distribution, determined through histograms and the Shapiro–Wilk test, are presented as medians with (interquartile range). The prespecified primary outcome was liver TG, and secondary outcomes were fasting and postprandial plasma glucose, insulin, and plasma TG concentrations. For the primary outcome, we used Wilcoxon signed rank tests with Bonferroni correction (n = 2) to test separately for effects between LCHF and CON and VLCD and CON, respectively. This approach was prespecified in the protocol because we hypothesized that liver TG content would be lower after both interventions than after CON, and power calculations (n = 12) were based on detecting 10% difference in liver TG content after LCHF and VLCD compared with after CON with 85% power and a 5% significance level. Thus, we did not power the study to detect differences between VLCD and LCHF. To account for potential failed ^1^H-MRS scans, we initially included 15 participants in the study. After conducting blinded quality assurance at study conclusion, we obtained complete ^1^H-MRS data for 10 participants. For other outcomes, exploratory comparisons between the 3 interventions were conducted using 1-way repeated measures analysis of variance (for normally distributed data) or the Friedman test (for non-normally distributed data). In cases of a statistically significant effect (*P* ≤ .05), post hoc paired t-tests or Wilcoxon signed rank tests were used for pairwise comparisons between LCHF and CON and VLCD and CON, respectively.

To assess postprandial differences, repeated measures mixed-effect models were employed, testing for differences between postprandial time of blood sampling (time) and diet (VLCD compared with CON and LCHF compared with CON). Time and diet were used as fixed parameters and subject as a random effect. Post hoc analysis as pairwise testing between each timepoint were applied in cases where the time × diet interaction was significant. Statistical analyses were performed in SPSS (version 29.0.1.0, IBM Corp, NY, USA) and GraphPad Prism (version 9, GraphPad, CA, USA).

## Results

### Participant Characteristics and Dietary Interventions

We included male and postmenopausal female individuals with overweight or obesity. According to the FIB-4 index and HbA_1c_, the participants had a low risk of liver fibrosis and did not have diabetes (Table 1).

**Table 1.** Participants characteristics measured at screening

	All (n = 15)	Males (n = 8)	Females (n = 7)
Age (years)	54 (47-58)	50 (42-57)	55 (51-60)
Body weight (kg)	92 (85-113)	109 (100-125)	87 (84-81)
BMI (kg/m^2^)	33 (31-34)	36 (32-40)	31 (31-33)
Abdominal circumference (cm)	108 (96-116)	116 (113-117)	96 (93-101)
HbA_1_c (mmol/mol)	38 (37-39)	38 (37-40)	38 (37-39)
ALAT (U/L)	21 (20-47)	43 (22-53)	20 (20-21)
ASAT (U/L)	26 (20-32)	26 (20-35)	22 (22-31)
FIB-4 index	0.97 (0.7-1.2)	0.96 (0.5-1.2)	1.03 (0.8-1.4)

Presented as mean ± SD.

Abbreviations: ALAT, alanine aminotransferase; ASAT, aspartate aminotransferase; BMI, body mass index; FIB-4 index, fibrosis index based on 4 factors; HbA_1c_, glycosylated hemoglobin.

The composition of the control diet (CON) reflected the dietary composition of the average Danish population (49E% carbohydrate, 16E% protein, and 35E% fat) (23). LCHF and VLCD were matched in absolute carbohydrate contents (∼60 g carbohydrates/day). Both diets also contained 20E% protein (hence differing in absolute values) and differed both in the absolute values and in energy percentage in the amount of fat to maintain isocaloric conditions for LCHF (same total energy content as CON) or hypocaloric conditions (2720/3347 kJ/day for females and males, respectively) for VLCD. Energy intakes during LCHF, VLCD, and CON are listed in Table 2. Notably, energy intake was reduced by 76 ± 1% during VLCD compared with LCHF (Table 2).

**Table 2.** Dietary composition and energy consumption

All (n 15)			
Dietary composition	VLCD	CON	LCHF
Energy intake (kJ)	2985 ± 88	12 633 ± 652	12 878 ± 626
**Relative intake**			
Protein, E%	20.8 ± 0.1	19.9 ± 0.1	19.9 ± 0.2
Carbohydrate, E%	36.1 ± 0.5	49.0 ± 0.1	8.1 ± 0.2
Fat, E%	40.2 ± 0.4	29.3 ± 0.1	72.1 ± 0.2
Saturated fatty acids, % of total fat	40.2 ± 1.5	32.6 ± 0.1	30.6 ± 0.2
Monounsaturated fatty acids, % of total fat	43.3 ± 1.1	44.7 ± 0.1	39.9 ± 0.2
Polyunsaturated fatty acids, % of total fat	16.5 ± 0.4	22.7 ± 0.1	29.5 ± 0.1
**Absolute intake**			
Protein, g	37 ± 1	148 ± 8	151 ± 8
Carbohydrate, g	63 ± 1	364 ± 19	60 ± 1.5
Dietary fiber, g	12 ± 0.4	42 ± 2	26 ± 1.3
Fat, g	32 ± 1	97 ± 5	245 ± 12
Cholesterol, mg	52 ± 2	128 ± 7	262 ± 14

Data are shown for the control diet (CON), very-low–calorie diet (VLCD), and isocaloric low-carbohydrate, high-fat diet (LCHF) per day. E%: percentage of total energy intake. Information on the fatty acid type was available for approximately 70-80% of the total fat in the intervention diets. Consequently, data for the fatty acid type are presented as a percentage of known fatty acids. Values are mean ± SE.

### Liver TG Content

**Figure 1.**
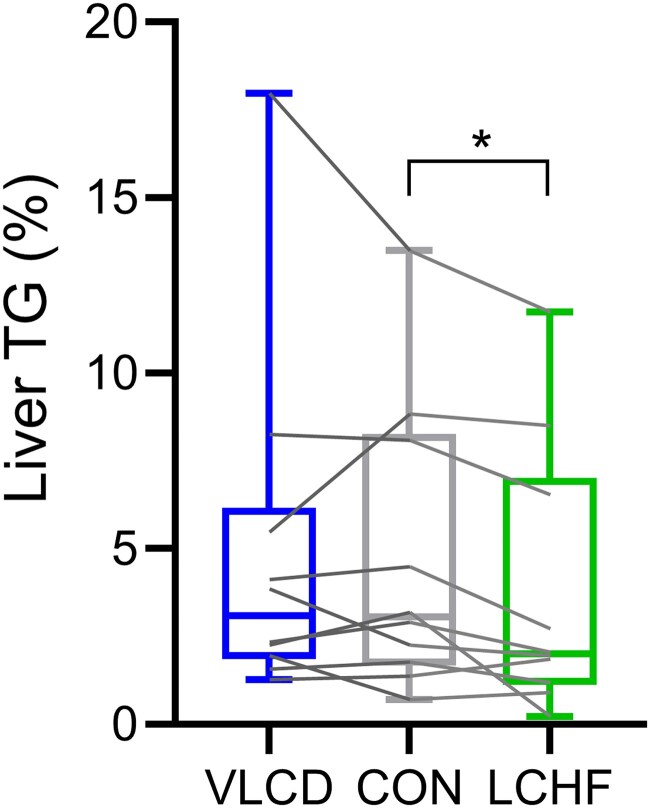
Liver triacylglycerol content. Liver triacylglycerol (TG) content (%) measured by ^1^H-magnetic resonance spectroscopy (^1^H-MRS) in the overnight fasted state after 2 days of control diet (CON), very-low calorie diet (VLCD), and isocaloric low-carbohydrate, high-fat diet (LCHF), respectively. Data are presented as medians with interquartile range. Wilcoxon signed rank test with Bonferroni correction was used to test for differences between CON and VLCD and between CON and LC. n = 10. *P < .05.

Figure 1 shows the liver TG content (%) measured by ^1^H-MRS in the overnight fasted state after 2 days of the 3 types of diet. Median liver TG content was 3.1% (range 0.7;12.8) after CON, 2.0% (range 0.2-11.5) after LCHF, and 3.1% (range 1.3-16.7) after VLCD. Liver TG content was −16% (IQR −34 to +4) after LCHF relative to after CON (*P* = .020). Conversely, liver TG content after VLCD did not differ from liver TG levels after CON (*P* = .519).

Fasting plasma concentrations of liver enzymes were similar after LCHF (30 ± 4 U/L and 23 ± 2 U/L for ALAT and ASAT, respectively) and VLCD (29 ± 4 U/L and 25 ± 2 U/L) when compared with after CON (31 ± 5 U/L and 24 ± 2 U/L).

### Bodyweight and Body Composition

After 2 days of CON diet, body weight was 98.8 ± 4.0 kg (Table 3). Body weight was 1.1% (1.0 kg) lower after LCHF (*P* = .006) and 1.8% (1.8 kg) lower after VLCD (*P* < .001). This was accompanied by a lower total fat mass after the LCHF (−0.5 kg; *P* = .024) and the VLCD interventions (−0.4 kg; *P* = .023). The relative body fat mass percent was similar after the diets. Lean mass was lower after the VLCD (−0.5 kg; *P* = .038) and similar after LCHF and CON. Visceral fat mass and total body water did not differ after the diets (Table 3).

**Table 3.** Body composition

n = 15	VLCD	CON	LCHF	*P* value 1-way ANOVA	*P* value VLCD vs CON	*P* value LCHF vs CON
Body weight, kg	97.0 ± 3.9	98.8 ± 4.0	97.8 ± 4.0	<.001	<.001	.006
BMI, kg/m^2^	32.2 ± 0.7	32.8 ± 0.7	32.4 ± 0.7	<.001	<.001	.004
Fat mass, kg	37.6 ± 1.6	38.3 ± 1.7	37.5 ± 1.6	.012	.013	.013
Body fat, %	39.0 ± 1.6	39.1 ± 1.5	38.7 ± 1.6	.530	—	—
Lean mass, kg	28.8 ± 1.8	29.2 ± 1.8	29.3 ± 1.8	.010	.038	.555
Visceral fat, L	4.3 ± 0.6	4.2 ± 0.6	4.2 ± 0.6	.152	—	—
Total body water, L	43.8 ± 2.3	44.2 ± 2.4	44.5 ± 2.4	.304	—	—

Measured by bioimpedance after 2 days of very-low calorie diet (VLCD), control diet (CON) and isocaloric low-carbohydrate, high-fat diet (LCHF). BMI, body mass index. Data are presented as means ± SE. n = 15. Comparisons between the 3 interventions were conducted using 1-way ANOVA (for normally distributed data) or the Friedman test (for non-normally distributed data). In cases of significant effect (*P* ≤ .05), post hoc t-tests or Wilcoxon Signed Rank tests were used for pairwise comparisons between LCHF and CON and VLCD and CON, respectively.

### Plasma Triacylglycerol, Fatty Acid, and Glycerol

Fasting plasma TG concentration was 48% lower after LCHF (*P* < .001) and 41% lower after VLCD (*P* < .001) than the CON diet, respectively (Table 4), resulting in a 36% lower tAUC of plasma TG during the meal test after LCHF (*P* < .001) and a 38% lower tAUC after VLCD (*P* < .001) (Table 4). The postprandial change from baseline (ΔTG_peak-fasting_) was similar after LCHF and CON (*P* = .779) and 40% lower after the VLCD vs CON (*P* = .023).

**Table 4.** Measures of glucose and lipid metabolism under conditions of overnight fasting and during a 4-hour mixed meal test after the dietary interventions

n = 15	VLCD	CON	LCHF	*P* value 1-way ANOVA	*P* value VLCD vs CON	*P* value LCHF vs CON
Fasting glucose mmol/L	4.7 ± 0.1	5.4 ± 0.1	5.2 ± 0.1	<.001	<.001	.033
tAUC_0-240_ glucose, mmol/L × min	1587 ± 38	1454 ± 39	1607 ± 51	<.003	.005	.004
iAUC_0-240_ glucose, mmol/L × min	454 ± 43	155 ± 33	365 ± 41	<.001	<.001	<.001
Fasting insulin, pmol/L	21 (17-36)	58 (47-93)	45 (28-57)	<.001	<.001	<.022
tAUC_0-240_ insulin, pmol/L × min	71 967 (51 657-99 940)	64 054 (54 098-78 319)	72 931 (51 574-10 6287)	.131	—	—
iAUC_0-240_ insulin, pmol/L × min	65 892 (46 736-94 111)	52 162 (40 209-64 971)	62 107 (45 922-95 571)	.134	—	—
Fasting C-peptide, pmol/L	385 (283-490)	690 (586-850)	604 (488-709)	<.001	<.001	.026
tAUC_0-240_ C-peptide, pmol/L × min	523 399 (445 339-606 776)	439 298 (371 543-510 491)	508 421 (421 136-602 591)	.005	.008	.021
iAUC_0-240_ C-peptide, pmol/L × min	443 265 (365 486-481 204)	296 014 (226 343-331 826)	384 323 (304 136-438 135)	<.001	<.001	<.001
HOMA2 IR	0.9 (0.6-1.0)	1.6 (1.3-1.9)	1.4 (1.1-1.6)	<.001	<.001	.025
HOMA-β	101 (84-109)	116 (102-141)	106 (98-126)	.003	.011	.173
Fasting C-peptide/insulin	17 ± 1	12 ± 1	14 ± 1	<.001	<.001	.012
IGI	531 (435-599)	787 (543-1197	598 (437-673)	.007	.006	.006
DI	551 (467-834)	513 (326-577)	421 (358-539)	.005	.135	.326
β-Hydroxybutyrate, µmol/L	0.34 ± 0.02	0.15 ± 0.02	0.23 ± 0.08	<.001	.005	.001
Fasting FA, µmol/L	720 ± 50	562 ± 32	564 ± 39	.003	.017	.968
tAUC_0-240_ FA, µmol/L × min	107 451 ± 11 448	64 773 ± 6384	78 879 ± 6538	<.001	<.002	.013
dAUC_0-240_ FA, µmol/L × min	−73 890 (−111 420; −54 615)	−72 461 (−94 538; −51 454)	−63 188 (−79 860; −43 508)	.533	—	—
Fasting TG, mmol/L	1.1 ± 0.1	1.9 ± 0.2	1.0 ± 0.1	<.001	<.001	<.001
tAUC_0-240_ TG, mmol/L × min	287 ± 24	494 ± 57	299 ± 28	<.001	<.001	<.001
ΔTG_peak-fasting_, mmol/L	0.23 ± 0.18	0.44 ± 0.30	0.46 ± 0.31	.010	.023	.779
Fasting glycerol, µmol/L	45 ± 5	35 ± 5	39 ± 5	.032	.021	.347
tAUC glycerol_0-240_, µmol/L × min	8664 ± 913	7099 ± 982	8780 ± 1123	.016	.013	.008
dAUC_0-240_ glycerol, µmol/L × min	−2160 ± 977	−1277 ± 763	−571 ± 631	.179	—	—

Data are presented following 2 days of control diet (CON), 2 days of a very-low-calorie diet (VLCD), and 2 days of an isocaloric low-carbohydrate, high-fat diet (LCHF). For normally distributed data, mean ± SE is presented, while non-normally distributed data are represented by median and interquartile range. n = 15. Comparisons between the 3 interventions were conducted using 1-way analysis of variance (for normally distributed data) or the Friedman test (for non-normally distributed data). In cases of significant effect (*P* ≤ .05), post hoc t-tests or Wilcoxon Signed Rank tests were used for pairwise comparisons between LCHF and CON and VLCD and CON, respectively.

Abbreviations: DI, disposition index; FA, fatty acid; IGI, insulinogenic index; tAUC; total area under the curve.

**Figure 2.**
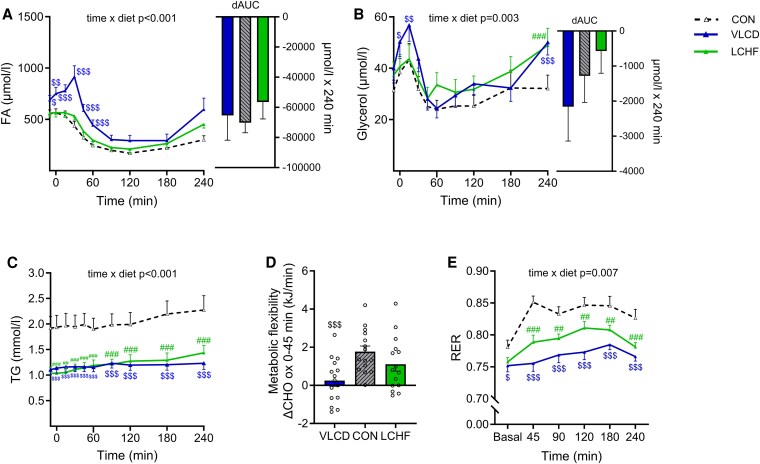
Postprandial lipid and substrate metabolism. Postprandial (4 hours) response a to liquid mixed meal test (600 kcal, 74 g carbohydrate, 24 g protein, 23 g fat) following 2 days of control diet (CON), 2 days of very-low–calorie diet (VLCD), or 2 days of isocaloric low-carbohydrate, high-fat diet (LCHF). (A) Plasma fatty acid (FA) concentration, (B) plasma glycerol concentration, (C) plasma triacylglycerol concentration, (D) metabolic flexibility, and (E) respiratory exchange ratio (RER). To assess postprandial differences, repeated measures mixed-effect models were employed, testing for differences between postprandial time of blood sampling (time) and diet (VLCD compared with CON and LCHF compared with CON). Time and diet were used as fixed parameters and subject as a random effect. Post-hoc analysis was applied in cases where the time × diet interaction was significant. Values are presented as means with standard errors. n = 15. #*P* < .05; ##*P* < .01; ###*P* < .001: LCHF significantly different from CON; $*P* < .05; $$*P* < .01; $$$*P* < .001: VLCD significantly different from CON.

Fasting plasma FA and glycerol concentrations were similar after LCHF and CON diets but were 40% (*P* = .017) and 56% (*P* = .021) higher, respectively, after the VLCD intervention (Fig. 2A and 2B and Table 4). tAUCs of plasma FA and glycerol were 30% and 34% higher after LCHF (*P* = .013 and *P* = .008, respectively) and 79% and 42% higher after VLCD (*P* = .002 and *P* = .013, respectively) relative to after the CON diet (Table 4). The postprandial suppression of plasma FA from fasting concentrations were similar (ΔFA_fasting–nadir_/FA_fasting_) after all diets (LCHF 76% [79-62], VLCD 76% [89-60], CON 78% [89-69]).

### Substrate Oxidation

After the CON diet, fasting RER was 0.78 ± 0.01 and tended to be lower after LCHF (0.76 ± 0.01; *P* = .081), whereas it was lower after the VLCD (0.75 ± 0.01; *P* = .035) (Fig. 2E). Fasting resting energy expenditure was similar after all diets (LCHF 6170 ± 471 kJ/day, VLCD 5708 ± 457 kJ/day, CON5901 ± 405 kJ/day). Fasting plasma β-hydroxybutyrate concentrations were 1.5-fold higher after the LCHF intervention (*P* = .001) and 2-fold higher after the VLCD intervention (*P* < .005) than after CON, respectively (Table 4). Metabolic flexibility tended to be lower after LCHF (1.1 ± 0.4 kJ/min, *P* = .086) and was markedly lower after VLCD (0.2 ± 0.3 kJ/min, *P* < .001) than after CON (1.8 ± 0.3 kJ/min) (Fig. 2D).

### Fasting Plasma Cholesterol Profiles

Fasting plasma total cholesterol concentrations averaged 5.5 ± 0.3 mmol/L after CON and were similar after the LCHF and VLCD diets (5.4 ± 0.3 mmol/L and 5.4 ± 0.3 mmol/L, respectively). Fasting plasma HDL cholesterol concentration averaged 1.4 ± 0.1 mmol/L after CON and was 8% higher following LCHF (1.5 ± 0.1 mmol/L, *P* = 0.023), and similar after VLCD (1.3 ± 0.1 mmol/L). Fasting plasma LDL cholesterol concentration was similar after LCHF (3.5 ± 1.7 mmol/L) and after CON diets (3.2 ± 0.2 mmol/L) but was 13% higher after VLCD (3.6 ± 0.3 mmol/L, *P* = .027). Fasting plasma VLDL cholesterol concentration was 52% lower after the LCHF (0.42 ± 0.04 mmol/L, *P* < .001) and 43% lower after the VLCD (0.47 ± 0.05 mmol/L, *P* < .001) than after the CON diet (0.87 ± 0.10 mmol/L).

### Glucose and Insulin Responses

**Figure 3.**
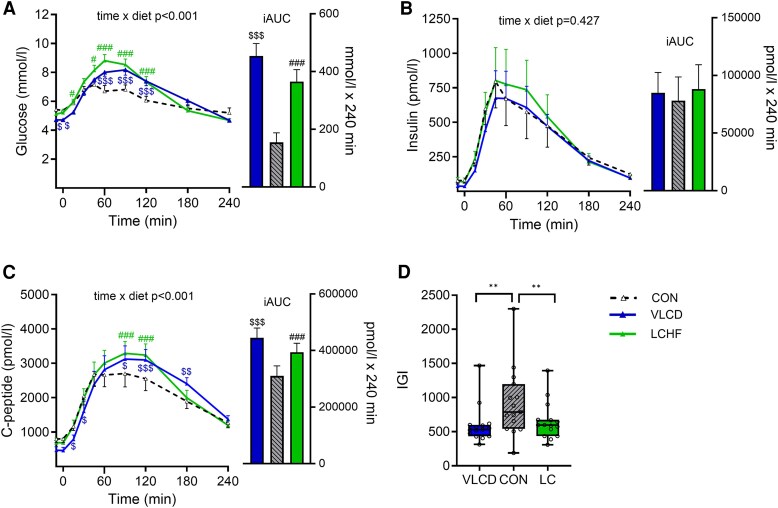
Postprandial glucose metabolism. Postprandial (4 hours) plasma glucose, insulin and C-peptide concentrations in response to a liquid mixed meal test (600 kcal, 74 g carbohydrate, 24 g protein, 23 g fat) following 2 days of control diet (CON), 2 days very-low calorie diet (VLCD), or 2 days of isocaloric low-carbohydrate, high-fat diet (LCHF). Values are presented as means ± SE. n = 15. (A) plasma glucose, (B) insulin, and (C) C-peptide concentrations, and (D) insulinogenic index (IGI) **P < .01. To assess postprandial differences, repeated measures mixed-effect models were employed, testing for differences between postprandial time of blood sampling (time) and diet (VLCD compared with CON and LCHF compared with CON). Time and diet were used as fixed parameters and subject as a random effect. Post hoc analysis was applied in cases where the time × diet interaction was significant. Paired t-tests were used to test for differences in iAUC between LCHF and CON and VLCD and CON, respectively. LCHF significantly different from CON #*P* < .05; ##*P* < .01; ###*P* < .001. VLCD significantly different from CON $*P* < .05; $$*P* < .01; $$$*P* < .001.

Fasting plasma glucose concentration was 5.4 ± 0.1 mmol/L after CON and was 0.2 ± 0.1 mmol/L lower after LCHF (*P* = .033) and 0.7 ± 0.1 mmol/L lower after VLCD (*P* < .001). Peak plasma glucose concentration after meal intake relative to fasting was higher after LCHF (+4.1 ± 0.3 mmol/L; *P* < .001) and VLCD (+3.8 ± 0.3 mmol/L; *P* < .001 than the CON (+2.4 ± 0.2 mmol/L), respectively. After meal intake, tAUCs of plasma glucose were 11% higher following the LCHF (*P* = .004) and 10% higher following VLCD (*P* = .005) relative to after CON (Table 4). When taking baseline values into account (iAUC), the postprandial plasma glucose concentrations were 2-fold higher after the LCHF diet (*P* < .001) and 3-fold higher after VLCD (*P* < .001) (Fig. 3A and Table 4).

Fasting plasma insulin concentrations were 19% lower after LCHF (*P* = .022) and 64% lower after VLCD (*P* < .001) than after CON (Fig. 3B and Table 4). tAUC and iAUC of plasma insulin concentration during the meal did not differ significantly after the 3 diets (Fig. 3B and Table 3). Fasting plasma C-peptide concentration was 16% lower after the LCHF (*P* = .026) and 46% lower after the VLCD intervention (*P* < .001) relative to after CON (Fig. 3C and Table 3). After meal intake, tAUC of plasma C-peptide were 14% higher after LCHF (*P* = .026) and 14% higher after VLCD (*P* = .008) (Table 4). Furthermore, plasma C-peptide levels (iAUC) were 34% higher after the LCHF intervention (*P* < .001) and 52% higher after the VLCD intervention (*P* < .001) (Fig. 3C and Table 4). The surrogate measure of insulin clearance in the fasted state (C-peptide/insulin) was 14% higher after the LCHF (*P* = .012) and 45% higher after the VLCD (*P* < .001) than after the CON diet (Table 4). HOMA-β was not impacted by the LCHF diet but was 16% lower after VLCD vs CON (*P* = .011), (Table 4). HOMA2-IR was 17% lower after LCHF (*P* = .025) and 44% lower after VLCD (*P* < .001) relative to after CON (Table 4). IGI was lower after both LCHF (*P* = .006) and VLCD (*P* = .006) vs CON), whereas the disposition index was similar after all diets (Table 4).

### Paracetamol Absorption Rates

**Figure 4.**
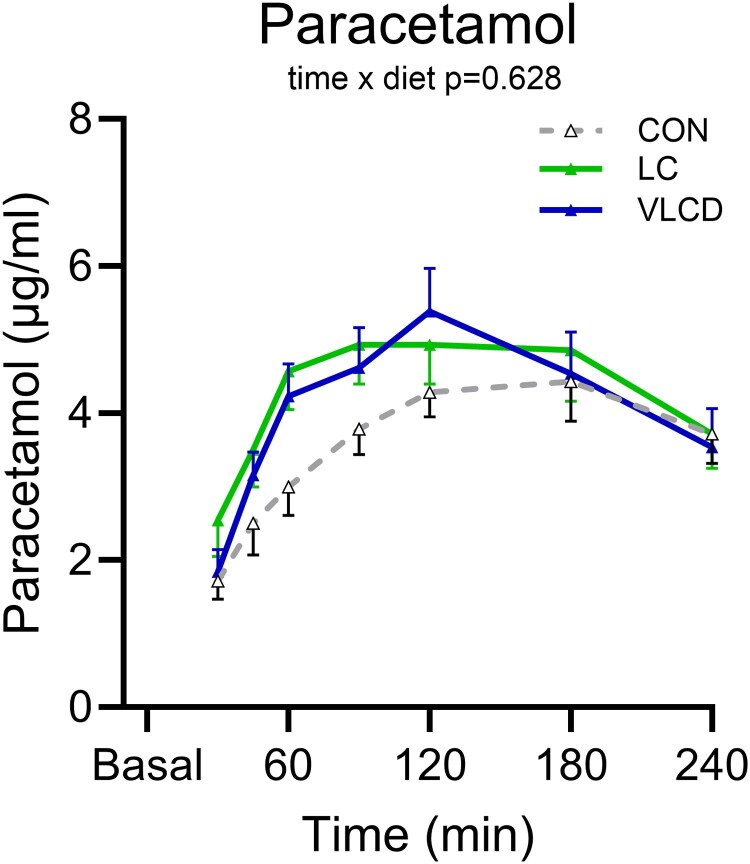
Paracetamol absorption. Postprandial (4 hours) serum paracetamol following 2 days of control diet (CON), 2 days very-low calorie diet (VLCD), or 2 days of isocaloric low-carbohydrate, high-fat diet (LCHF). Values are presented as means ± SE. To assess postprandial differences, repeated measures mixed-effect models were employed, testing for differences between postprandial time of blood sampling (time) and diet (VLCD compared with CON and LCHF compared with CON). Time and diet were used as fixed parameters and subject as a random effect. Post hoc analysis were applied in cases where the time × diet interaction was significant. n = 15.

Neither time to peak nor the postprandial concentrations curves for plasma paracetamol differed after diets (Fig. 4).

### Ad Libitum Meal

Ad libitum meal energy intake was comparable after all diets (LCHF 2313 ± 346 kJ, VLCD 2025 ± 293 kJ, CON 2335 ± 346 kJ).

## Discussion

To the best of our knowledge, this is the first study investigating the immediate response in liver TG content and whole-body metabolism to a 2-day fixed carbohydrate-restricted diet (60 g/day) under both hypocaloric conditions and isocaloric conditions, with the latter maintaining energy content through high-fat intake. Furthermore, no studies have examined whether carbohydrate restriction, differing in energy provision, impacts postprandial glucose metabolism differently once carbohydrates are reintroduced. In our study, only isocaloric, but not hypocaloric, carbohydrate restriction led to a lower liver TG content. This could support a positive role of sustaining energy provision through increased dietary fat content for liver TG reduction, as opposed to the effect of carbohydrate reduction per se. Calorie restriction–induced weight loss is a well-established stimulus to lower liver TG content in individuals with obesity (24, 25). Interestingly, we did not observe a difference in liver TG content following the VLCD in our study contrasting findings from Kirk et al (2009), in which a hypocaloric (4603 kJ/day) carbohydrate-restricted diet (carbohydrates; <50 g/day) lowered liver TG content within 2 days (5). This could be attributed to a greater reduction in energy provision during the VLCD intervention in our study (76% restriction; corresponding to 2985 ± 88 kJ intake/day). Along this line, complete fasting for 24 to 36 hours has been demonstrated to increase both fasting plasma FA levels and liver TG content (26, 27). In contrast, a study applying a diet with energy provision of 2510 kJ/day (∼60 carbohydrates/day), comparable to that of VLCD in our study, demonstrated a 30% decrease in liver TG content within 7 days concurrently with a weight loss of 3.9 ± 0.2 kg (6). This could suggest that the longer duration of the diet intervention and/or the more pronounced weight loss, of 3.9 kg compared with 1.8 kg weight loss in our study during the VLCD intervention, is of importance for the regulation of liver TG content. While the VLCD was insufficient to impact on liver TG content after 2 days in our study, the LCHF diet, wherein the carbohydrate restriction was substituted with fat to maintain isocaloric conditions, lowered liver TG content by 16%. This aligns with another acute study showing decreased liver TG content after 24 hours of an isocaloric low-carbohydrate (4E%, ∼30 g), high-fat diet (7).

Both dietary interventions were associated with higher FA oxidation and ketogenesis in our study, as indicated by lower RER values and elevated fasting circulating β-hydroxybutyrate concentrations, suggesting marked alterations in the hepatic metabolism. However, the higher FA oxidation and ketogenesis were apparently not sufficient to lower liver TG content after the VLCD intervention. This could be caused by a differential effect of the 2 diets on adipose tissue lipolysis with substantially increased concentrations of circulating FAs after the VLCD intervention, but not after the LCHF diet, suggesting a potentially higher FA influx to the liver specifically after the VLCD. Adipose tissue lipolysis–derived FA in the circulation is known to be a determinant of liver TG content (28). Thus, the increase in dietary fat during isocaloric carbohydrate restriction in our study appears to ensure that the energy provision is maintained avoiding high adipose tissue lipolysis derived FA influx from the circulation as seen in the hypocaloric carbohydrate-restricted condition.

In the overnight fasted state circulating TG levels most often reflect VLDL-TG secretion rate (29) and is positively associated with liver TG content (30). However, in our study, fasting plasma TG was lowered—not just after the LCHF diet as expected from liver TG data—but also after the VLCD intervention. The lower plasma TG following the VLCD is therefore not likely to be caused directly by reduced liver TG content and reduced VLDL-TG secretion but could be speculated to involve enhanced VLDL-TG clearance. This is supported by findings showing higher VLDL-TG clearance after low- compared with high-carbohydrate diets (31) and the facts that insulin lowers (32), while calorie restriction for 2 days increases (33), muscle lipoprotein lipase activity. This could potentially imply an increased capacity for clearing VLDL-TG during carbohydrate and calorie restriction.

The LCHF encompassed favorable alterations in lipid and glucose metabolism in the overnight fasted state. Hence, fasting insulin and glucose concentrations were lower along with increased estimates of fasting hepatic insulin clearance and lower HOMA2-IR index suggesting higher hepatic insulin sensitivity. These beneficial metabolic effects mimic observations after longer-term low-carbohydrate, high-fat dietary regimens in both patients with type 2 diabetes (11, 16-18) and in healthy individuals (34). Interestingly, we also observed favorable alterations in fasting glucose metabolism and HOMA2-IR after the VLCD intervention despite that liver TG content remained unchanged. We speculate that the VLCD diet might introduce a shift in the composition of liver lipids, rather than a reduction in total TG content. A lowering of intermediate lipid species as ceramides and diacylglycerols, which are known to impair hepatic insulin signaling (35, 36), could potentially contribute to the improved HOMA2-IR, although this remains speculative.

In contrast, despite very different energy provision and fat content between the LCHF and the VLCD intervention the postprandial plasma glucose concentrations after a fixed carbohydrate-rich meal were increased after both diets. Correspondingly, glycemic control was impaired on the day following 1 day of an isocaloric low-carbohydrate (5E%), high-fat (75E%) diet in a previous study (37). Additionally, impaired glucose handling during an oral glucose tolerance test (38, 39) or a mixed meal test (40) has also been reported after 3 days to 4 weeks of a low-carbohydrate, high-fat intake. The mechanisms underlying the impaired postprandial glucose handling may be caused by a combination of several factors. We found that the early β-cell responses (evaluated by the IGI) to the mixed meal ingested after the LCHF and VLCD interventions were impaired. This could contribute to higher postprandial glucose concentrations, since an effective early insulin response is crucial for maintaining glucose tolerance (41). We demonstrated an increase in plasma FA after the VLCD intervention, which previously has been suggested to impair β-cell function and peripheral insulin sensitivity (42). Accordingly, increased plasma FA concentrations may have contributed to the impaired β-cell function (eg, lower HOMA-β and IGI) and decreased postprandial glucose tolerance after the VLCD intervention but is less explanatory for the impaired postprandial glucose tolerance after the LCHF diet, where plasma FA levels were not elevated. Moreover, although the impaired postprandial glucose tolerance could suggest inflexibility in β-cells to initially adapt to the higher carbohydrate load during the meal test, it might also reflect a β-cell adaptation in response to improved hepatic insulin sensitivity (43), since the disposition index was unchanged after both diets. Of note, it is debated whether β-cell adaptation relates to insulin sensitivity in the liver or the periphery (44), and it may differ in fasting and postprandial conditions (44, 45). Moreover, both an impaired postprandial inhibition of hepatic glucose production and a lower peripheral glucose disposal could in theory contribute to higher postprandial glucose concentrations, which we cannot clarify in the present study, where we did not apply glucose tracers. In support of a peripheral rather than a hepatic defect, we found the surrogate of hepatic insulin resistance (HOMA2-IR) to be lower after both diets in accordance with a previous study showing that a 36-hour fasting period improved hepatic insulin action despite concomitantly impairing whole-body insulin action (43). Also, the well-described “glucose-fatty acid cycle” by Randle and co-workers (46) proposes that during high FA availability, fat oxidation is favored at the expense of glucose oxidation, which results in intramyocellular accumulation of glucose metabolic intermediates lowering the gradient for glucose to be taken up across the muscle cell membrane, in turn diminishing glucose uptake in muscle and hence increasing circulating glucose concentrations (46). Such a substrate-competitive glucose–FA cycle could contribute to the impaired glucose handling after carbohydrate restriction. However, in our study postprandial circulating FA levels were found to be elevated only after the VLCD intervention. Also, impairment in the ability to switch to glucose oxidation during meal-induced insulin stimulation—referred to as metabolic flexibility—was also most markedly impaired after the VLCD intervention. Metabolic flexibility describes the ability to shift between fat and glucose oxidation depending on nutrient availability and/or hormonal signals, such as transitioning from primarily fat oxidation during fasting to greater reliance on glucose oxidation in the fed or insulin-stimulated state. Thus, while substrate competition could explain impaired glucose handling via reduced peripheral glucose-induced glucose disposal after the VLCD intervention, this model is not fully explaining the post-LCHF situation. Importantly, the impaired postprandial glucose response after the diets might be a temporary metabolic response, since neither low-carbohydrate, high-fat diets nor low-calorie diets impairs insulin sensitivity measured by hyperinsulinemic euglycemic clamp after longer interventions (6, 47-49). Also, further studies are needed to investigate whether the impaired postprandial glucose response persists during subsequent meals, providing a better understanding of how quickly the body readapts when carbohydrates are reintroduced.

Lastly, in this study we could not detect significant differences in gastric emptying rates using the paracetamol absorption test, although previous studies using gold standard scintigraphic measures of gastric emptying may support adaptation of consuming a diet enriched in fat with subsequently accelerated emptying of a standard mixed meal which might also contribute to increased postprandial glucose concentration (50).

It is worth considering that adherence to continuous energy-restricted or low-carbohydrate diets have proven almost certainly imperfect, when individuals are allowed free-living conditions without dietary control (15, 51, 52) prompting considerations of alternative approaches. However, the observed increase in postprandial plasma glucose concentrations in this study may question the real-world metabolic efficacy of dietary regimens such as intermittent fasting (eg, “the 5:2 diet”) (53) and intermittent carbohydrate restriction (54) as well as also imperfectly adhered low-carbohydrate, high-fat continuous dietary regimens. Although it is also possible that adaption to consistent shifts in macronutrients availability, potentially enhancing metabolic flexibility and substrate utilization would occur during longer term intermittent diets. Nevertheless, from a liver-centric perspective, intermittent isocaloric carbohydrate restriction with high dietary fat provision may offer advantages over intermitting fasting regimens for reducing liver TG levels.

In the present study, body weight was 0.5 kg lower after LCHF than after CON in our randomized crossover design. This was surprising, since energy intake was strictly matched between these interventions. In comparison, body weight was 1.8 kg lower after VLCD than after CON. 24-hour energy expenditure has been shown to be higher on low-carbohydrate, high-fat diets than high-carbohydrate diets using metabolic chambers or double-labelled water measurements (55-57); however, we did not observe differences in metabolic rate in the fasted state using indirect calorimetry. Other studies have also demonstrated weight losses during low-carbohydrate, high-fat diets despite energy matching (11, 15-17, 40). In the present study, all food was provided for the participants, but nevertheless we cannot completely rule out that weight loss may result from incomplete consumption of scheduled meals. That aside, the reduction in weight and fat mass seems unlikely to be solely attributed to energy deficit, as this would require rather large energy deficit of approximately 16 MJ over the 2 days (based on the required energy deficit per unit weight loss of 32.2 MJ/kg (58)). Instead, changes in glycogen stores and less water binding as a result hereof appears more likely to fluctuate over short periods and mediate reduced weight.

This study has certain limitations that should be considered when interpreting the results. First, the intervention periods were relatively short, and the sample size was limited. Notably, the randomized crossover design allowed each participant to serve as their own control, which increases statistical power. Second, the study was designed to investigate acute, short-term metabolic responses to dietary interventions and does not address the effects of long-term dietary changes. Importantly, the short intervention periods were necessary to isolate the metabolic effects of macronutrient composition, as extending the duration would introduce confounding effects from weight loss in the hypocaloric VLCD condition. As such, the findings provide mechanistic insight into short-term adaptations in liver TG content, glucose, and lipid metabolism, and should be interpreted accordingly.

In conclusion, 2 days of isocaloric carbohydrate restriction with high dietary fat provision resulted in lower liver TG content compared with after a control diet, which was not observed after 2 days of similar carbohydrate restriction under very-low calorie conditions in males and postmenopausal females with overweight or obesity. These findings illustrate the dynamic regulation of liver fat content within days and highlight the beneficial role of substituting carbohydrates with fat to maintain energy balance. Furthermore, this emphasizes the importance of controlling diet before assessing liver TG content in both clinical and research settings, as liver TG levels reflect the individual's current metabolic state and is influenced by the recent dietary macronutrient composition. Both carbohydrate restricted diets encompassed favorable alterations in lipid and glucose metabolism in the overnight fasted state. However, the postprandial plasma blood glucose concentrations after reintroducing dietary carbohydrates were impaired after both isocaloric and hypocaloric carbohydrate restriction, which partially could be ascribed to lower early-phase insulin secretory response.

## Data Availability

Data described in the manuscript, will be made available upon reasonable request.

## Clinical Trial Information

Clinicaltrials.gov ID (NCT05643521, registered November 22, 2022).

## Abbreviations

^1^H-MRS: ^1^H-magnetic resonance spectroscopy
ALAT: alanine aminotransferase
ASAT: aspartate aminotransferase
AUC: area under the curve
BMI: body mass index
CON: control diet
dAUC: decremental area under the curve
E%: energy-%
FA: fatty acid
HDL: high-density lipoprotein
iAUC: incremental area under the curve
IGI: insulinogenic index
LCHF: isocaloric low-carbohydrate, high-fat diet
LDL: low-density lipoprotein
RER: respiratory exchange ratio
TG: triacylglycerol
tAUC: total area under the curve
VLCD: very-low–calorie diet
VLDL: very-low-density lipoprotein
